# Management of displaced humeral surgical neck fractures in daily clinical practice: hanging does not re-align the fracture

**DOI:** 10.1007/s00402-022-04545-8

**Published:** 2022-07-16

**Authors:** Reinier W. A. Spek, Lotje A. Hoogervorst, Michaëla E. C. Elias, Ruurd L. Jaarsma, DirkJan H. E. J. Veeger, Job N. Doornberg, Paul C. Jutte, Michel P. J. van den Bekerom

**Affiliations:** 1 Department of Orthopaedic Surgery, Flinders University and Flinders Medical Centre, Flinders Dr, Bedford Park, Adelaide, 5042 Australia; 2 Department of Orthopaedic Surgery, University of Groningen and University Medical Centre, Groningen, The Netherlands; 3 Department of Orthopaedic Surgery, OLVG, Amsterdam, The Netherlands; 4 Department of Orthopaedic Surgery, Department of Biomedical Sciences, Medical Decision Making, Leiden University Medical Centre, Leiden, The Netherlands; 5 Department of Biomechanical Engineering, Delft University of Technology, Delft, The Netherlands; 6 Department of Human Movement Sciences, Faculty of Behavioral and Movement Sciences, Vrije Universiteit Amsterdam, Amsterdam Movement Sciences, Amsterdam, The Netherlands

**Keywords:** Surgical neck fractures, Proximal humerus fracture, Non-operative management, Re-alignment, Boileau classification, Radiographic evaluation

## Abstract

**Introduction:**

It is unclear if the collar and cuff treatment improve alignment in displaced surgical neck fractures of the proximal humerus. Therefore, this study evaluated if the neckshaft angle and extent of displacement would improve between trauma and onset of radiographically visible callus in non-operatively treated surgical neck fractures (Boileau type A, B, C).

**Materials and Methods:**

A consecutive series of patients (≥ 18 years old) were retrospectively evaluated from a level 1 trauma center in Australia (inclusion period: 2016–2020) and a level 2 trauma center in the Netherlands (inclusion period: 2004 to 2018). Patients were included if they sustained a Boileau-type fracture and underwent initial non-operative treatment. The first radiograph had to be obtained within 24 h after the initial injury and the follow-up radiograph(s) 1 week after trauma and before the start of radiographically visible callus. On each radiograph, the maximal medial gap (MMG), maximal lateral gap (MLG), and neck-shaft angle (NSA) were measured. Linear mixed modelling was performed to evaluate if these measurements would improve over time.

**Results:**

Sixty-seven patients were included: 25 type A, 11 type B, and 31 type C fractures.

The mean age (range) was 68 years (24–93), and the mean number (range) of follow-up radiographs per patient was 1 (1–4). Linear mixed modelling on both MMG and MLG revealed no improvement during follow-up among the three groups. Mean NSA of type A fractures improved significantly from 161° at trauma to 152° at last follow-up (*p-*value = 0.004).

**Conclusions:**

Apart from humeral head angulation improvement in type A, there is no increase nor reduction in displacement among the three fracture patterns. Therefore, it is advised that surgical decision-making should be performed immediately after trauma.

**Level of clinical evidence:**

Level IV, retrospective case series.

**Supplementary Information:**

The online version contains supplementary material available at 10.1007/s00402-022-04545-8.

## Introduction

In displaced surgical neck fractures of the humerus, it is not well understood which fracture patterns would respond best to non-operative treatment and which ones would require surgical fixation [1]. If non-operative treatment is chosen, patients are advised to wear a collar and cuff with their arm in internal rotation and the humeral shaft in line with the humeral head. In this position, while holding the body upright, traction is generated due to gravity, allowing the shaft to realign with the proximal humerus [2]. However, re-alignment may not occur in each type of fracture and if it fails, surgical management can be required to avoid mal- or non-union.

**Fig. 1:**
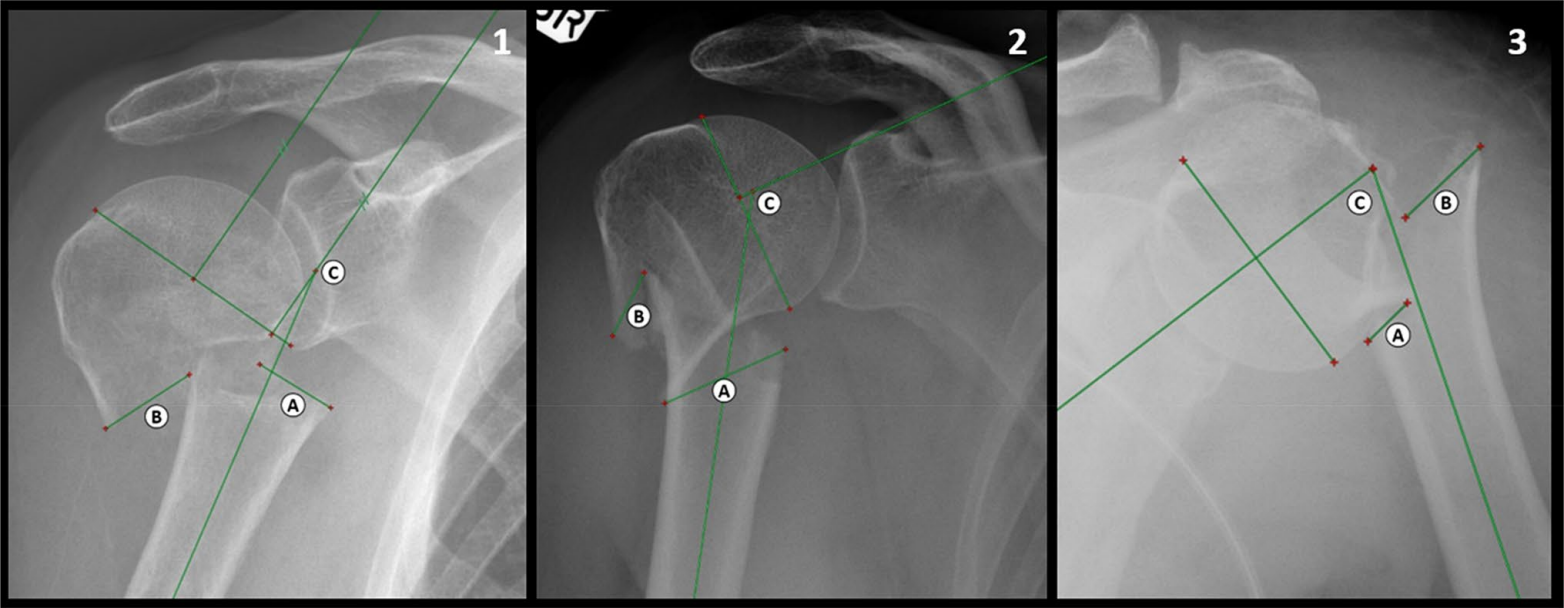
1 = type A (partial medial shaft displacement with valgus angulation), 2 = type B (entire medial and ventral shaft displacement without humeral head tilt), 3 = type C (lateral shaft displacement with varus angulation). Three parameters were measured on each radiograph: A = maximal medial gap, B = maximal lateral gap, C = neck-shaft angle

Besides biomechanical forces (e.g., muscles and bone-on-bone friction) when wearing a collar and cuff, there may be a relationship between fracture pattern and alignment. To date, few studies have evaluated radiographic outcomes in non-surgically treated proximal humerus fractures. One study revealed that radiographic angulation on lateral views after 1 week could predict outcomes in minimally displaced proximal humerus fractures [3]. However, it remained unclear if the collar and cuff treatment would improve angulation and shaft translation in fractures with ≥ 1 cm of displacement. A French study conducted by Boileau et al. recently classified surgical neck fractures into three categories: type A, B and C (Table 1, Fig. 1). Considering the surgical nature of this work, we hypothesized that hanging down the arm in a collar and cuff (as applied in current clinical practice) would not re-align these three fracture patterns [4]. The aim of this study was to assess (1) if the neck-shaft angle and extent of displacement would improve between trauma and onset of radiographically visible callus in non-operatively treated surgical neck fractures (Boileau type A, B, C), and (2) if there would be a difference in displacement and humeral head tilt between type A, B or C.

**Table 1:** Fracture patterns according to Boileau’s classification

	Humeral shaft translation	Humeral head position
Type A	Partially medial	Valgus
Type B	Entirely medial and/or ventral	Neutral
Type C	Partially lateral	Varus

## Material and methods

### Setting and study design

This retrospective imaging study was carried out in a level 2 trauma center in the Netherlands and a level 1 trauma center in Australia. Ethical approval was received in both centers in compliance with their local institutional review boards.

### Screening

In the Dutch Hospital, patients were included between January 1, 2004, and June 30, 2018. The inclusion period from the Australian trauma center was from March 1, 2016, to July 31, 2020. All surgical neck fractures within this period were screened and categorized according to Boileau’s classification system [4]. Screening and classification were performed independently and in duplicate by the first three authors. Discrepancies were resolved by discussion. If consensus could not be achieved, one of the surgeons in our author group was consulted.

### Study population

Patients (≥ 18 years) with type A, B or C (according to Boileau’s classification) isolated displaced surgical neck fracture were included in the study [4, 5]. Patients with pathologic surgical neck fractures, undeterminable humeral head angulation on trauma radiographs, concomitant fractures (large Hill-Sachs lesions, greater tuberosity fractures with footprint defects, humeral shaft-, clavicle-, and acromion fractures), and patients who underwent surgery before day 8 after initial trauma were excluded. Patients were required to have an anteroposterior (AP) radiograph obtained within 24 h after the initial injury and at least one follow-up AP radiograph while following a non-operative treatment protocol. follow-up radiographs needed to be available at least one week after the initial trauma and before the start of the radiographically visible callus (Supplement 1).

### Classification

Boileau et al. classified surgical neck fractures into three categories (Table 1, Fig. 1): (type A): partial medial shaft displacement with valgus angulation of the humeral head (shoulder adductor muscles, predominantly the pectoralis major- and latissimus dorsi muscle, pull the shaft medially resulting in humeral head tilt to the contralateral side), (type B) entire medial and ventral shaft displacement without humeral head tilt, (type C) lateral shaft displacement with varus angulation of humeral head (shoulder abductor muscles, acromial part of deltoid and biceps brachii, pull the shaft laterally and supraspinatus muscle pulls head in further varus tilt). As the original classification article did not specify displacement, we used a displacement cut-off of ≥ 25% of the humeral shaft diameter. Patients were categorized as type B if they had complete shaft translation in any direction (as opposed to the medial and anterior translation described by Boileau et al.). Dorsal or ventral head angulation was not taken into account: if a patient had medial shaft translation with valgus and dorsal head deformity, the patient was still categorized as type A. If fracture patterns contradicted Boileau’s criteria, they were categorized into the miscellaneous category ‘’unclassifiable’’ and excluded for further analysis. For example, if there was partial shaft translation without humeral head angulation.

### Hospital treatment protocol and variables

Routine assessment of patients with a displaced surgical neck fracture in both hospitals included physical examination and radiographic imaging. If non-operative treatment was followed, patients were provided with a collar and cuff with the arm in adduction (elbow and forearm act as a weight to provide traction) and shoulder movements were allowed as tolerated by pain. Surgical decision-making was based on patient comorbidities and fracture patterns. The following variables were collected for each patient: age, gender, date of hospital admission, side of the fracture, days from initial injury to first trauma radiograph, type of treatment, type of surgical treatment, time between injury and surgery, presence of comminution, number of follow-up radiographs, and time from trauma to each radiograph.

### Outcome measures

As the Boileau classification is based on deformities in the frontal plane, only radiographic parameters were measured on anteroposterior (AP) radiographic views. The following parameters were measured on each trauma and follow-up radiograph: maximal medial gap (MMG), maximal lateral gap (MLG), and neck-shaft angle (NSA) (Fig. 1). The MMG was defined as the maximal distance between the medial tip of the surgical neck and the edge of the fracture on the inferior humeral head on the medial side, the MLG as the maximal distance between the lateral tip of the surgical neck and the edge of the fracture on the inferior humeral head on the lateral side. MMG, MLG and gap were all evaluated in millimeter (mm) and measured between both outer cortices. The NSA was calculated by drawing a line through the middle of the humeral shaft (bisector), the anatomic neck and a line perpendicular to the anatomic neck. The NSA represented the angle between the bisector and the line perpendicular to the anatomic neck. Measurements of radiographs in the Dutch Hospital were performed using Agfa Health Care (Agfa-Gevaert Group, Mortsel, Belgium) and in the Australian Hospital with RadiAnt DICOM Viewer (Medixant, Poznan, Poland) [6]. All measurements were performed by one assessor (first or second author).

### Statistical analysis

Data analyses were performed using IBM SPSS software version 27 (IBM Corp., Armonk, N.Y., USA). Categorical baseline characteristics were presented in numbers with percentages and continuous baseline variables with mean and range depending on the distribution. To assess if MML, MMG and NSA would improve over time in each Boileau type, linear mixed modelling (LMM) was conducted. This model was run separately for each fracture pattern and each outcome measure and included time as a co-variate with MML, MMG or NSA as s dependent variable. A random intercept was used, and time slopes were assumed to be fixed. Linear mixed modelling (LMM) was again performed to determine if there was a difference of MML and MMG between the three fracture types. This model contained MML or MLG as dependent variables, time as co-variate and Boileau classification as a factor. Fixed effects estimate (fee) was reported together with a 95% confidence interval (CI) and *p-*value. A *p-*value less than 0.05 was considered significant. Further to this, the MMG, MLG and NSA at trauma were compared between in- and excluded patients using an independent samples t-test (Supplement 2).

## Results

**Fig. 2:**
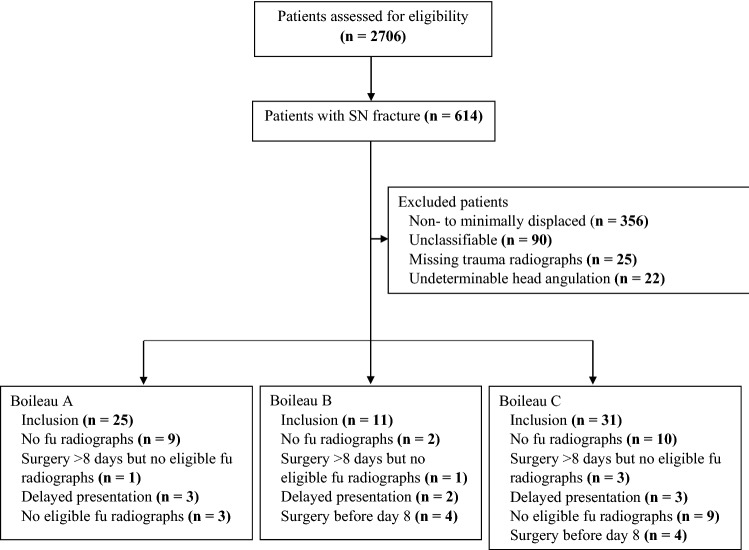
Breakdown of patients screened for a Boileau fracture. *SN* surgical neck, *fu* follow-up

A total of 2706 patients were screened for eligibility of which 614 patients had a displaced or undisplaced surgical neck fracture (most common reason for exclusion was the presence of a concomitant proximal humerus fracture such as a tuberosity fracture). Amongst these 614 surgical neck fractures, we identified 121 patients with a Boileau fracture: 41 type A, 20 type B, and 60 type C fractures. Only 1 (2.4%) patient in type A underwent surgery without eligible follow-up radiographs, 5 patients (25.0%) in type B and 7 patients (11.9%) in type C. After assessment against exclusion criteria a cohort of 67 patients was included for further analysis: 25 with type A, 11 with type B, and 31 with type C (Fig. 2). Mean age (range) of the cohort was 68.4 years (24–94), the majority were females (62.7%), and the mean number (range) of follow-up radiographs per patient was 1.3 (1–4). Surgical intervention was mostly performed in patients with type B fractures (36.4%) and surgical neck comminution did not differ between the three groups (Table 2).

**Table 2:** Baseline demographics

	All (*n* = 67)	A (*n* = 25)	B (*n* = 11)	C (*n* = 31)
Age (years)	70 (24–93)	78 (42–93)	78 (59–83)	62 (24–91)
Gender				
Female	42 (63%)	20 (80%)	10 (91%)	12 (39%)
Male	25 (37%)	5 (20%)	1 (9%)	19 (61%)
Days to presentation	0 (0–1)	0 (0–1)	0 (0–0)	0 (0–1)
Hospital				
Dutch	45 (67%)	17 (68%)	8 (73%)	20 (65%)
Australian	22 (33%)	8 (32%)	3 (27%)	11 (36%)
Right sided fracture	35 (52%)	18 (72%)	5 (46%)	12 (39%)
Comminuted fracture	12 (18%)	5 (20%)	2 (18%)	5 (16%)
Surgical management	12 (18%)	5 (20%)	4 (36%)	3 (10%)
ORIF	9 (13%)	4 (16%)	2 (18%)	3 (10%)
Nail	3 (5%)	1 (4%)	2 (18%)	0 (0%)
Days until surgery	20.5 (12–200)	19 (12–32)	18 (15–36)	55 (30–200)
Radiographs per patient	1 (1–4)	1 (1–3)	1 (1–2)	1 (1–4)
Days to fu radiograph(s)	14 (8–134)	14.5 (8–72)	11 (8–31)	15 (8–134)

Data is presented as median (range) or number (%).*Fu* follow-up, *ORIF* open reduction and internal fixation

Overall, there was a minimal effect and no significant improvement over time for a maximal medial and maximal lateral gap in each fracture type. Mean maximal medial gap in type A fractures changed from 11 mm at trauma to 10 mm at ≥ 22 days follow-up (fee: 0.004, 95% CI: − 0.06 to 0.07, *p-*value = 0.89), in type B fractures from 29 mm at trauma to 35 mm at ≥ 22 days follow-up (fee: 0.125, 95% CI: − 0.36 to 0.61, *p-*value = 0.59), and in type C fractures from 14 to 10 mm at ≥ 22 days follow-up (fee: − 0.003, 95% CI: − 0.09 to 0.08, *p-*value = 0.94) (Table 3).

**Table 3:** Displacement (maximal medial gap) over time per fracture type

	A	B	C
Trauma	11.3 (2.1–27.9)	29.1 (13.7–43.8)	13.6 (3.5–31.1)
8–14 days	14.5 (0.0–30.1)	31.3 (10.6–44.7)	12.9 (0.0–38.3)
15–21 days	14.1 (2.7–25.9)	27.6 (18.7–41.2)	12.4 (3.4–25.5)
≥ 22 days	9.9 (3.6–14.5)	35.1 (29.9–40.3)	9.9 (0.0–26.2)
	*p-*value = 0.89	*p-*value = 0.59	*p-*value = 0.94

The maximal medial gap (distance between the medial tip of the surgical neck and medial fracture edge on the humeral head) was presented as the mean (range) in millimeter. *p-*values were obtained from linear mixed modelling with a maximal medial gap as a dependent variable and time as co-variate

Mean maximal lateral gap in type A was 14 mm at trauma and 13 mm at ≥ 22 days follow-up (fee: 0.012, 95% CI: − 0.05 to 0.07, *p*-value = 0.71). MLG in type B improved from 23 to 20 mm (fee: 0.126, 95% CI: − 0.22 to 0.47, *p-*value = 0.44) and in type C from 9 to 6 mm (fee: − 0.022, 95% CI: − 0.09 to 0.05, *p-*value = 0.51): trauma *versus* ≥ 22 days follow-up, respectively (Table 4).

**Table 4:** Displacement (maximal lateral gap) over time per fracture type

	A	B	C
Trauma	13.5 (5.2–27.7)	22.9 (11.4–41.4)	8.5 (2.0–27.0)
8–14 days	18.4 (4.0–34.7)	26.4 (14.3–33.5)	9.7 (0.0–30.8)
15–21 days	16.0 (1.9–29.2)	31.3 (26.9–38.2)	6.2 (0.0–12.7)
≥ 22 days	13.3 (3.6–26.3)	19.8 (10.8–28.8)	6.4 (0.0–14.3)
	*p-*value = 0.71	*p-*value = 0.44	*p-*value = 0.51

The maximal lateral gap (distance between the lateral tip of the surgical neck and lateral fracture edge on the humeral head) was presented as mean (range) in millimeter. *p-*values were obtained from linear mixed modelling with a maximal medial gap as dependent variable and time as co-variate

**Fig. 3:**
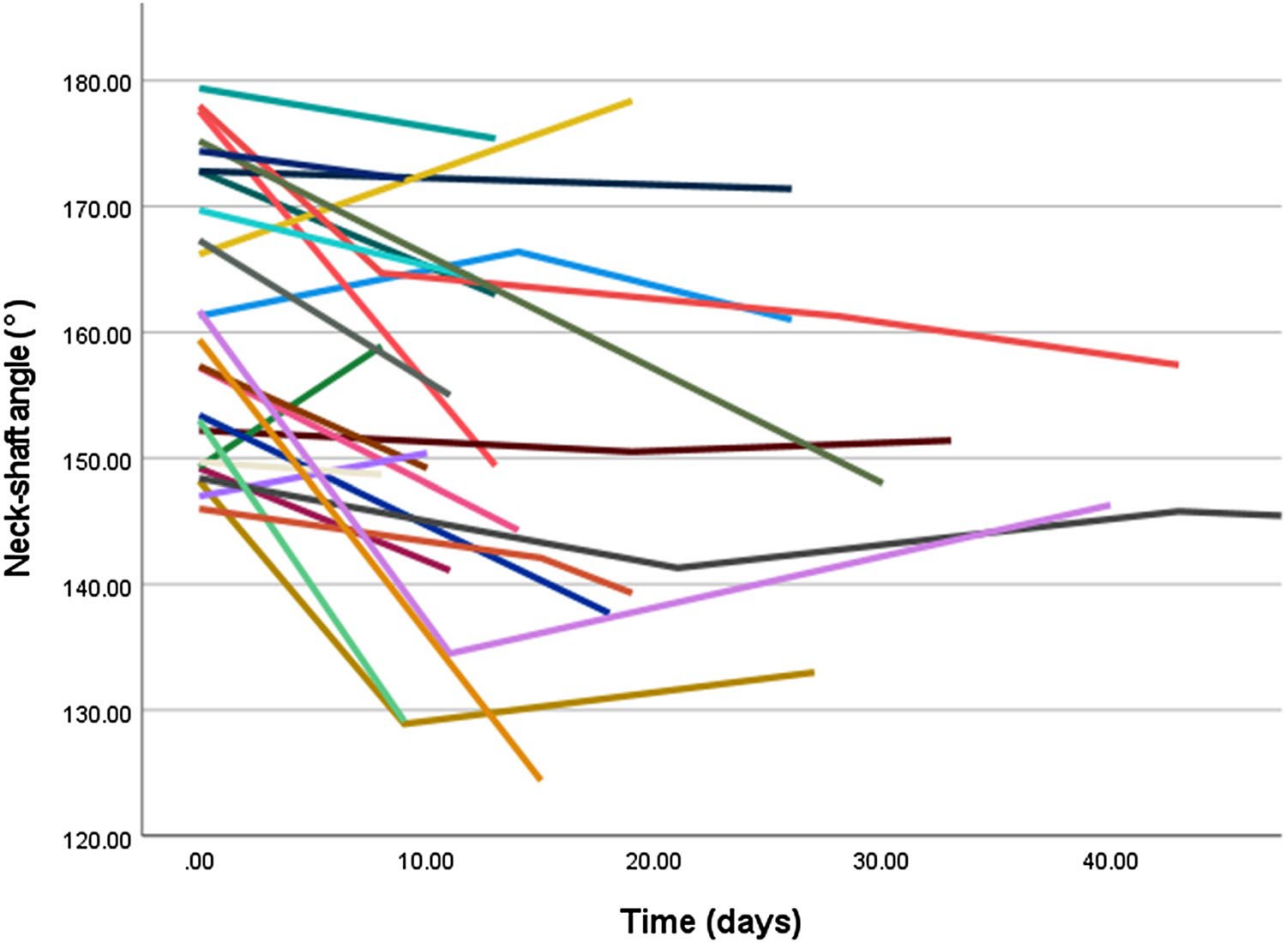
Multiple line graph of neck-shaft angle over time within type **A**. Each color represents a patient

**Fig. 4:**
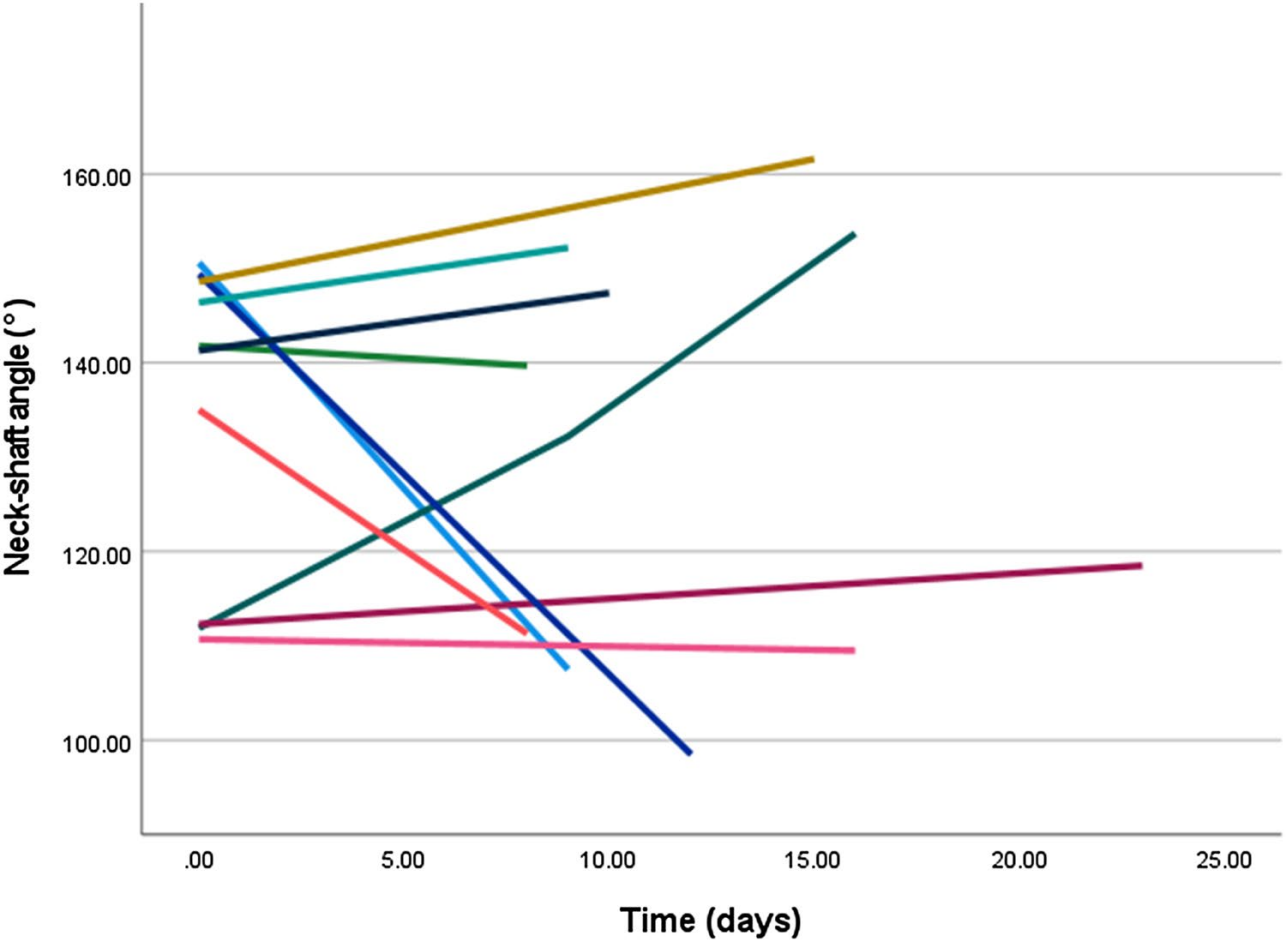
Multiple line graph of neck-shaft angle over time within type **B**. Each color represents a patient

**Fig. 5:**
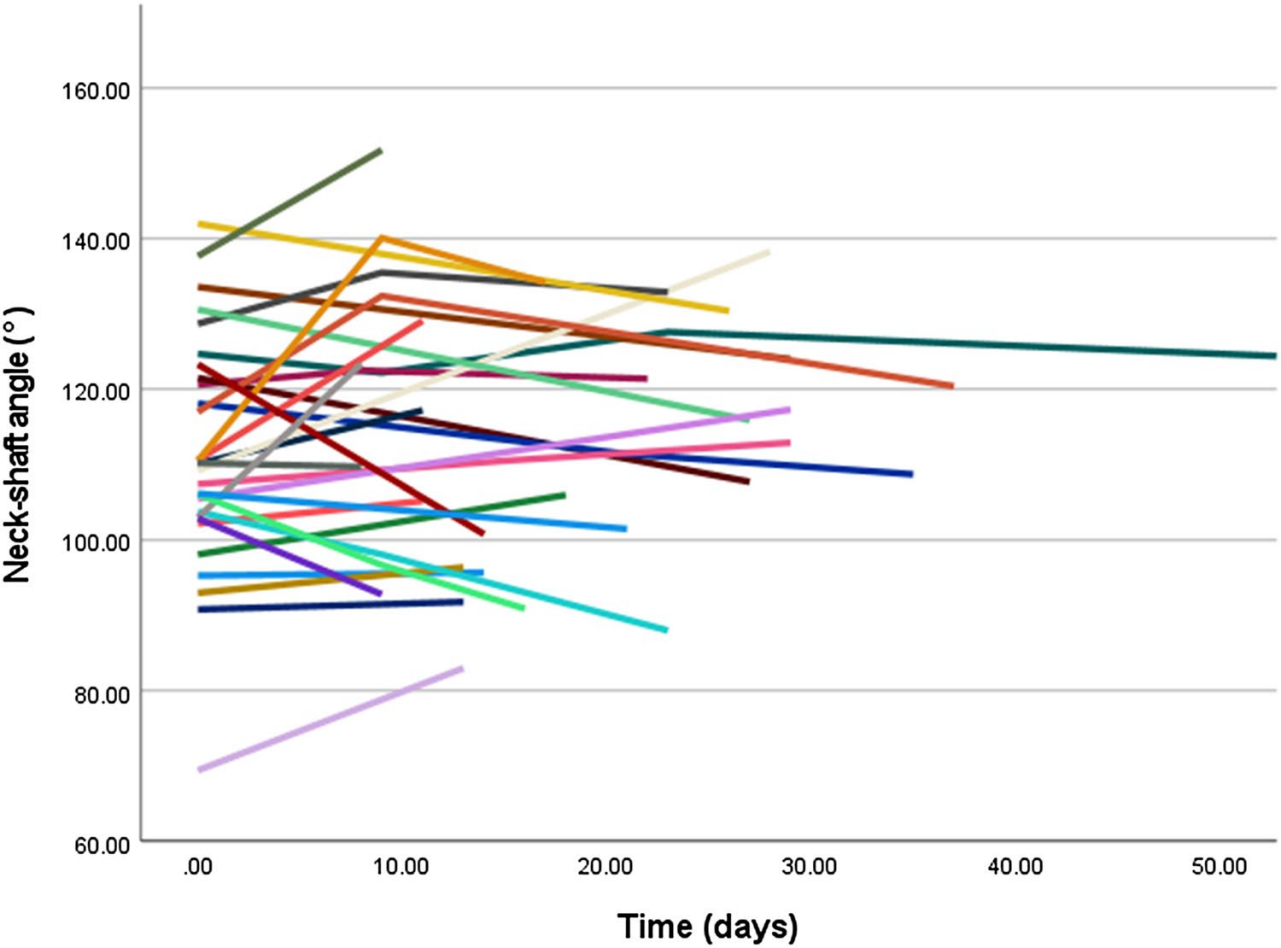
Multiple line graph of neck-shaft angle over time within type **C**. Each color represents a patient

**Fig. 6:**
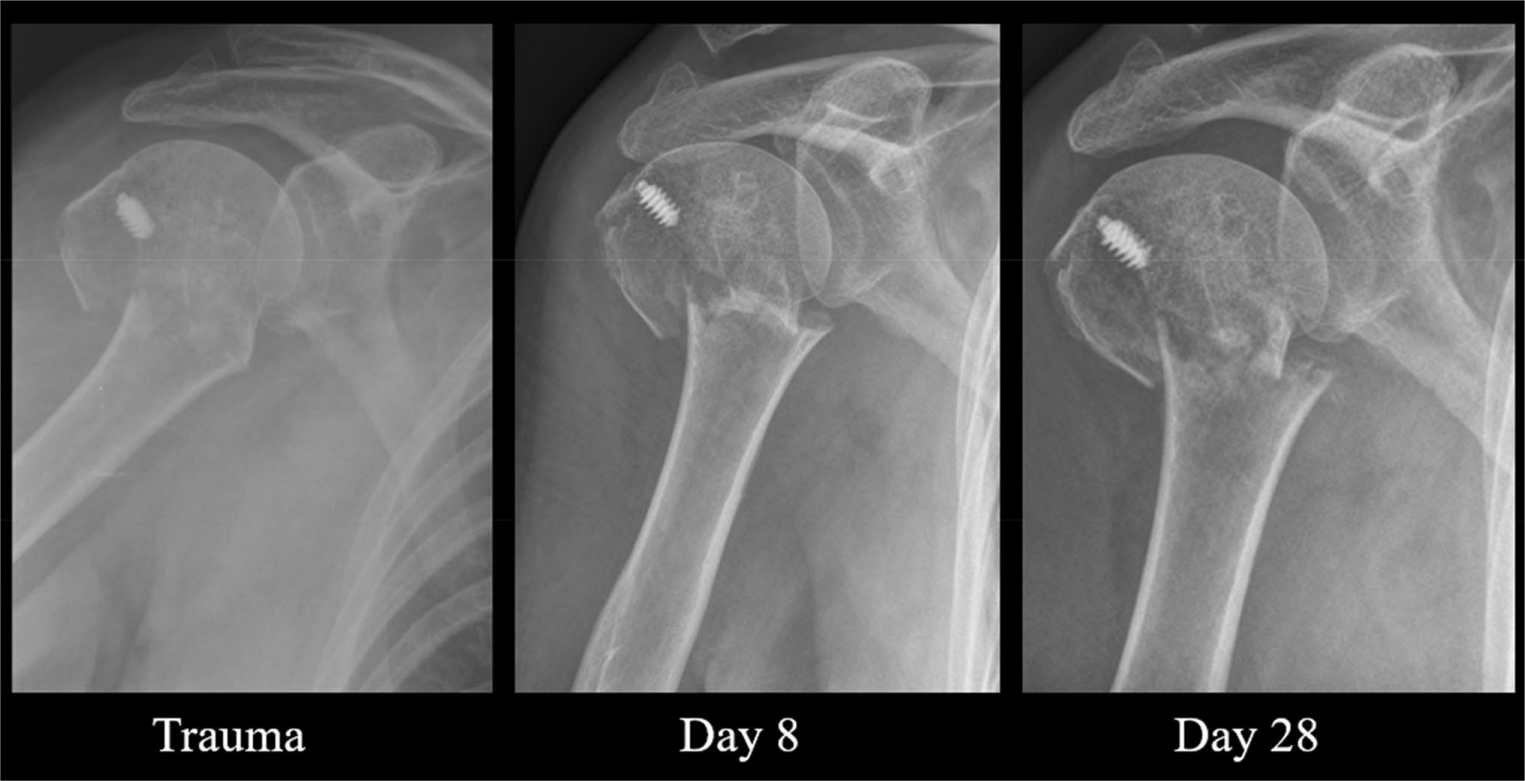
Radiographic follow-up of a type A fracture. Trauma: NSA = 178°, MMG = 5.6 mm, MLG = 10.1 mm. Day 8: NSA = 165°, MMG = 8.5 mm, MLG = 8.4 mm. Day 28: NSA = 161°, MMG = 10.3 mm, MLG = 11.2 mm

**Fig. 7:**
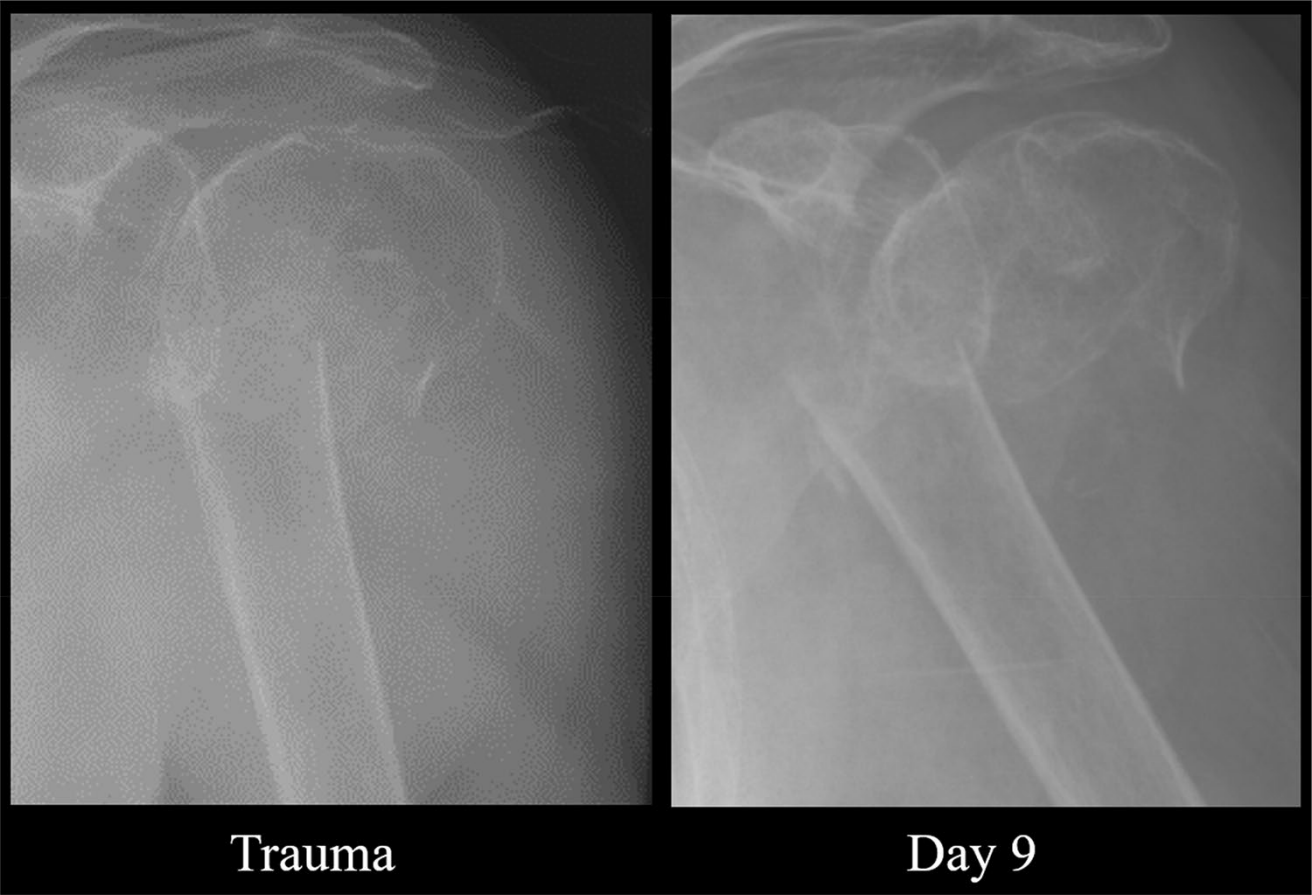
Radiographic follow-up of a type B fracture. Trauma: NSA = 146.4°, MMG = 24.9 mm, MLG = 18.6 mm. Day 9: NSA = 152.2°, MMG = 34.4 mm, MLG = 33.5 mm

**Fig. 8:**
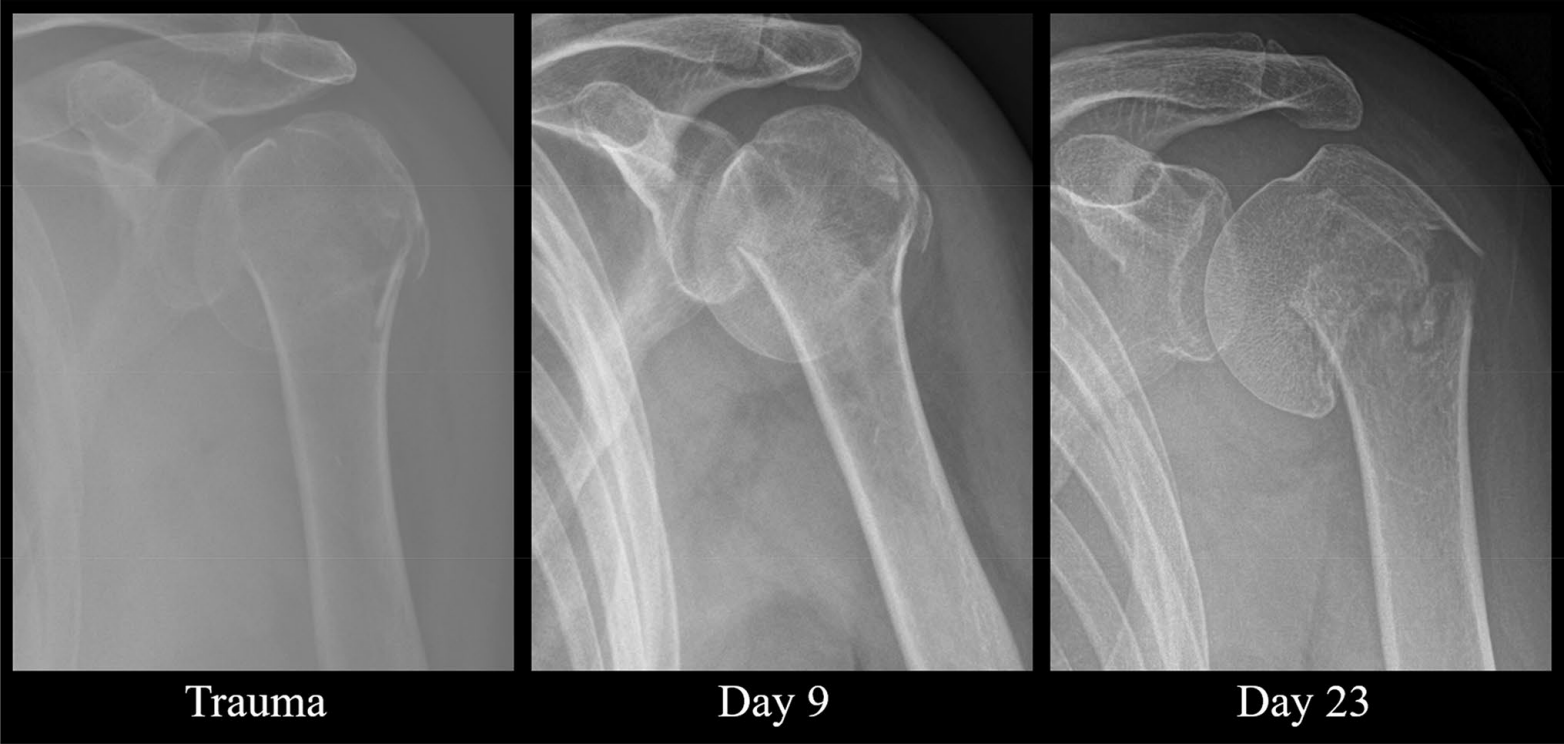
Radiographic follow-up of a type C fracture. Trauma: NSA = 103.8°, MMG = 28.1 mm, MLG = 2.1 mm. Day 9: NSA = 98.1°, MMG = 36.7 mm, MLG = 0.0 mm. Day 23: NSA = 88.0°, MMG = 18.1 mm, MLG = 14.3 mm

Except for type A fractures, neck-shaft angle did not improve significantly over time. Mean neck-shaft angle of type A improved from 161° to 152° at the last follow-up time frame. LMM revealed a significant relationship of NSA over time with a corresponding fee of − 0.28 (95% CI: − 0.46 to − 0.09, *p-*value = 0.004) (Fig. 3). For type B fractures, the mean NSA at trauma was 135° and the head remained in a neutral position until callus was visible on radiographs (fee: − 0.30, 95% CI: − 1.55 to 0.96, *p-*value = 0.62) (Fig. 4). Humeral head of type C remained in varus deformity during follow-up moments (fee: 0.01, 95% CI: − 0.12 to 0.13, *p-*value = 0.93) (Table 5) (Figs. 5, 6, 7, 8).

**Table 5:** Neck-shaft angle over time per fracture type

	A	B	C
Trauma	161.1 (146.0–179.4)	134.8 (110.7–150.6)	111.7 (69.4–142.0)
8–14 days	152.7 (128.9–175.4)	127.0 (98.5–152.2)	112.9 (83.0–151.8)
15–21 days	144.8 (124.4–178.4)	141.6 (109.5–161.6)	109.1 (90.9–134.2)
≥ 22 days	151.9 (133.0–171.4)	118.5 (118.5–118.5)	119.4 (88.0–138.3)
	*p-*value = 0.004	*p-*value = 0.62	*p-*value = 0.93

Data is presented as mean (range) in degrees. *p*-values were obtained from linear mixed modelling with neck-shaft angle as a dependent variable and time as co-variate

## Discussion

Besides establishing actual re-alignment forces when wearing a collar and cuff, evaluating the relationship between fracture patterns and alignment is relevant for clinical decision-making and determining follow-up trajectories of patients. Although we could not control for collar and cuff positioning, patient behavior, and compliance during management, we postulated that hanging down the arm in a collar and cuff would not re-align the three fracture patterns described by Boileau et al. [4]. In short, we found that apart from valgus head tilt improvement in type A, there was no significant increase nor reduction in displacement among all three Boileau types.

No improvement in the maximal medial gap and the maximal lateral gap was observed within the three fracture patterns. Biomechanically this can be explained by the trade-off between friction, gravity and forces exerted by shoulder muscles. Boileau’s fracture types are translated medially or laterally, so a force to the contralateral side may be required for reposition. Most likely the effect of gravity and or muscle activity in the collar and cuff is insufficient to reposition the humeral shaft, and due to muscular atrophy muscle strength also decreases over time [7]. However, studies are lacking on shoulder girdle- and pectoralis major muscle activity during immobilization. Natural traction force is determined by the gravity of the humerus and surrounding soft tissue which is governed by the weight of the arm. Assuming that patients are compliant and that their collar and cuff instructions were adequate, this natural traction force is apparently not sufficient to relocate the humerus shaft below the humeral head. It should also be considered that repositioning of the shaft requires counteracting forces that are exerted by muscles, tendons, fascia, and friction of bone-on-bone.

Interestingly, neck-shaft angle improved among type A fractures. Re-activation of the supraspinatus muscle during healing may explain this finding but the role of muscle activation during healing of this fracture is unknown. Another theory is that the resolving hematoma contributes (partially) to the head angle restoration. Fracture hematomas in proximal humerus fractures originate from the medial bone arteries and can subluxate the shoulder inferiorly due to accumulation in the glenohumeral joint [8–10]. Therefore, fracture hematomas are more likely to be located on the medial aspect of the humeral head rather than the lateral side. Resolution of hematoma will change the angle of the humeral head, resulting in restoration of traction from the rotator cuff muscles. This restoration might recover the original kinematic balance of the shoulder complex. In type B fractures, no significant NSA improvement was noted over time. Considering that the head is completely separated from the shaft, the forces of rotator cuff muscles are balanced and therefore the head remains in an anatomic position.

Confined to the limits of this study and an unknown quantity of traction, our findings suggest that radiographic re-alignment of type A, B and C fractures should not be expected to improve in clinical practice while managing patients with collar and cuff. Surgical decision-making should therefore be taken upon trauma, in contrast to greater tuberosity fractures where follow-up radiographs could change treatment strategy [11]. Surgical fixation may be required in patients with NSAs ≥ 160°, so since our data suggest that varus head deformity does not seem to improve over time, surgeons should be aware of this indication [12]. It should be stressed that non-operative treatment could still be valuable considering the high rate of complications in surgically treated patients [13]. For this reason, current guidelines need improvement and a better understanding of the biomechanical concept of this treatment. Further research is required into the actual amount and duration of natural traction provided by collar and cuff over a day using an instrumented collar and cuff construction to quantify the traction. We also advise further evaluating the optimal length of immobilization and activity of shoulder girdle muscles while the arm is immobilized and carrying out daily life activities. Additionally, this study should be repeated in a prospective design with patient-reported outcomes and follow-up radiographs at fixed time points. An interobserver study should be carried out to assess the reliability of the Boileau classification to see if it can be incorporated as a subclassification of Neer’s two-part fractures [14–17].

There are several shortcomings: first, compliance and collar and cuff instructions in this cohort were unknown. Incorrect collar and cuff positioning may not provide adequate natural traction, so this could have been the case in some patients. Second, the level of activity and general condition of patients were not collected. In bed-bounded patients, for example, natural traction on the fracture is lacking (bisector of the humeral shaft does not point downwards). However, most patients were included from the level 2 trauma center which does not treat polytrauma patients. Third, functional outcomes measures were not included, and selection bias may have been introduced as only a limited number of patients had eligible follow-up radiographs and some patients underwent surgery. Fourth, this classification system has not been evaluated in other studies so far and no sample size calculation was performed. However, effect sizes of displacement derived from linear mixed modelling were negligible and even the upper bound and lower bound of the 95% CIs did not exceed 1 mm. Therefore, a clinically relevant improvement is unlikely. Fifth, dorsal humeral head tilt was not considered when classifying the fractures and we included patients with a type B fracture if they had entire medial or ventral displacement. Sixth, analyses were not adjusted for internal or external rotation of the shoulder at trauma and during follow-up radiographs. However, due to pain, it is unlikely that trauma radiographs were taken with the arm in external rotation and radiographers are trained to obtain follow-up radiographs concordantly. Seventh, radiographs were not re-measured by a second researcher so we could not provide the reliability of the measurements. Despite these limitations, it should be acknowledged that collar and cuff treatment as applied in current orthopaedic clinical practice was evaluated and that our results are applicable to a relatively small sample of surgical neck fractures: Boileau fractures comprise only one-fifth of all surgical neck fractures.

## Conclusion

Apart from valgus head tilt improvement in type A, there is no significant increase nor reduction in displacement among all three Boileau types. One may argue that before the radiographically visible callus, there is no effect of hanging on re-alignment of the fracture in the frontal plane. In clinical practice, findings can be used for expectation management of patients and may indicate that re-alignment begins after the onset of callus formation. We advise that surgical decision-making should be performed immediately after trauma.

## Supplementary Information

Below is the link to the electronic supplementary material.

**Supplementary file1** (DOCX 102 KB)
