# Supplementary material for: Management of displaced humeral surgical neck fractures in daily clinical practice: hanging does not re-align the fracture
Source: Arch Orthop Trauma Surg. 2022 Jul 16;143(6):3119–28. doi: 10.1007/s00402-022-04545-8 (PMC10191947; doi:10.1007/s00402-022-04545-8)
Supplement: Supplementary file 1 — Supplementary file1 (DOCX 102 KB) [file 402_2022_4545_MOESM1_ESM.docx]

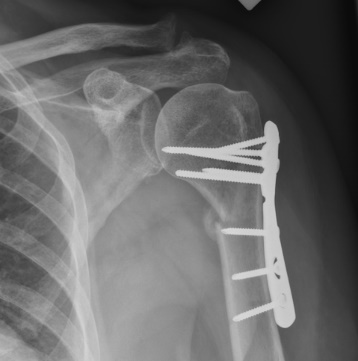

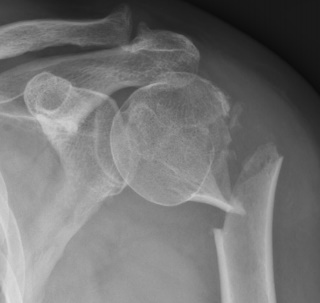


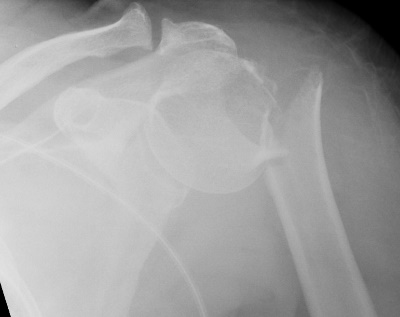


Eligible follow-up radiographs

Trauma

Day 8

Callus on radiograph

**Supplement 1** Outline of eligible follow-up radiographs. This example covers the follow-up of a type C fracture. follow-up radiographs must have been taken between day 8 post trauma and start of radiographically visible callus or surgical fixation.

|  | **Supplement 2** Comparison between in- and excluded patients for MMG, MLG and NSA | | | |  |
| --- | --- | --- | --- | --- | --- |
|  |  | **A** | **B** | **C** |  |
|  | MMG incl. | 11.3 ± 7.6 | 29.1 ± 10.2 | 13.6 ± 8.7 |  |
|  | MMG excl. | 9.1 ± 5.4 | 23.1 ± 10.0 | 16.0 ± 8.7 |  |
|  | *p-*value | 0.31 | 0.21 | 0.30 |  |
|  | MLG incl. | 13.5 ± 5.8 | 22.9 ± 8.7 | 8.5 ± 5.4 |  |
|  | MLG excl. | 15.8 ± 6.1 | 23.3 ± 8.5 | 9.8 ± 6.4 |  |
|  | *p-*value | 0.24 | 0.92 | 0.40 |  |
|  | NSA incl. | 161.1 ± 11.3 | 134.8 ± 15.8 | 111.7 ± 15.4 |  |
|  | NSA excl. | 159.0 ± 12.5 | 136.7 ± 13.7 | 114.4 ± 14.8 |  |
|  | *p-*value | 0.60 | 0.78 | 0.49 |  |
|  | MMG and MLG (millimeters) are presented in means ± standard deviation and NSA (degrees) in means ± standard deviation. Abbreviations: incl., included patients; excl., excluded patients; MMG, maximal medial gap; MLG, maximal lateral gap; NSA, neck-shaft angle | | | | |
